# Double Fortified Salt Containing Iodine and Folic Acid and Triple Fortified Salt Containing Iodine, Folic Acid, and Vitamin B_12_ Are Acceptable to Ethiopian Women of Reproductive Age

**DOI:** 10.1002/fsn3.70012

**Published:** 2025-01-30

**Authors:** Biniyam Tesfaye, Masresha Tessema, Charles D. Arnold, Tadesse Kebebe, Tesfaye Zeru, Teshome Assefa, Meseret Woldeyohannes, Getachew Tollera, Marinus Koning, Homero Martinez, Christine M. McDonald, Kenneth H. Brown

**Affiliations:** ^1^ Ethiopian Public Health Institute Addis Ababa Ethiopia; ^2^ Department of Nutrition and Institute for Global Nutrition University of California, Davis Davis California USA; ^3^ ReachAnother Foundation Bend Oregon USA; ^4^ Nutrition International Ottawa Canada; ^5^ University of California San Francisco San Francisco California USA

**Keywords:** folic acid, salt acceptability, salt fortification, vitamin B_12_

## Abstract

Recognizing the broader accessibility of iodized salt compared to wheat flour, the Ethiopian government is considering fortification of iodized salt with folic acid, and possibly vitamin B_12_, to address the high incidence of neural tube defects (NTDs) in Ethiopia. To prepare for this program, we assessed practices related to edible salt procurement and consumption, and the acceptability of novel salts fortified with iodine and folic acid (double‐fortified salt, DFS) or iodine, folic acid, and vitamin B_12_ (triple‐fortified salt, TFS) compared to iodized salt (IS). We surveyed 840 women of reproductive age in urban (Addis Ababa) and rural (Gimbichu Woreda) areas to describe their salt preferences and practices and used a three‐point hedonic scale to evaluate the sensory acceptability of fine and coarse forms of DFS, TFS, and IS for color, taste, aroma, texture and overall acceptability. We found that women were the primary salt purchasers (72.8% rural, 97.4% urban) for household. Rural women preferred coarse salt (76.1%), whereas urban women preferred fine salt (42.4%) or both types (30.0%). All salts received favorable mean sensory ratings (≥ 2.1), with fine salt preferred for all sensory characteristics (*p* < 0.05). Fine IS (2.8 ± 0.5) was rated slightly higher than DFS (2.6 ± 0.8) and TFS (2.5 ± 0.8) (*p* < 0.05). Rural women were more willing to purchase DFS (79.2%) and TFS (73.0%) than urban women (65.0% DFS, 63.8% TFS). In conclusion, DFS and TFS are highly acceptable in Ethiopia, with women being the key target for their introduction. Mandatory fortification of both coarse and fine salts is recommended to maximize reach.

## Graphical abstract

This study assessed salt procurement, utilization, and the acceptability of double‐fortified salt (DFS) with iodine and folic acid and triple‐fortified salt (TFS) with iodine, folic acid, and vitamin B12 among Ethiopian women of reproductive age. Sensory evaluations revealed high acceptability, particularly among rural women, highlighting the potential for fortification programs to improve public health outcomes related to neural tube defects.

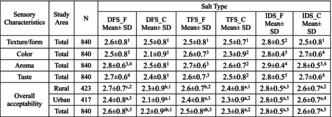

## 1 Introduction

Ethiopia experiences a high prevalence of neural tube defects (NTDs), with estimates varying depending on the study and detection method: Ssentongo et al. (2022) reported 60 cases per 10,000 births, Bitew et al. (2020) reported 63.3 cases per 10,000 children, and Tirsit et al. (2023) stated an ultrasound‐based prevalence of 166 cases per 10,000 fetuses. By contrast, the average global prevalence of NTDs is approximately 19 per 10,000 live birth (Blencowe et al. 2018). Maternal folate insufficiency is the primary risk factor for NTDs (Bailey and Hausman 2018), although maternal vitamin B_12_ deficiency has also been proposed as a possible contributor (Molloy et al. 2009). Both folate insufficiency and vitamin B_12_ deficiency occur frequently in Ethiopia, where, according to the 2016 nationally representative survey, a staggering 74.6% of women of reproductive age (WRA) have folate insufficiency (EPHI 2019), defined as red blood cell folate concentration < 748 nmol/L (Pfeiffer 2016) and 15% of WRA have vitamin B_12_ deficiency (serum cobalamin concentration < 203 pg/mL) (Ethiopian Public Health Institute (EPHI) 2016). In response to this situation, the Ethiopian Ministry of Health has proposed several intervention strategies to control folate insufficiency, including large scale food fortification with folic acid to improve maternal folate status and thereby reduce the risk of NTDs (EPHI 2019).

The primary vehicles for large‐scale folic acid fortification globally are wheat flour, maize flour, and rice (Allen et al. 2006). However, the reach of fortifiable cereals is relatively low in Ethiopia. Nationally, only 30% of the population has access to fortifiable wheat flour, with 45%, reach in urban areas and just 23% in rural areas (EPHI 2023). Production of fortifiable flour other than wheat flour is not common in Ethiopia, where the vast majority of cereals, including the native grain teff, are milled in small‐scale community mills, which account for an estimated 65% of the total milling capacity (Minot et al. 2015). Moreover, the country is not self‐sufficient in wheat, rice or maize production. Approximately 30% of wheat (Abu Tefera 2022), 76% of rice (Alemu 2022) and 0.01% maize grains are imported in the form of food aid (USAID 2017). By contrast, 100% of the population has access to iodized salt (EPHI 2023), and Ethiopia is self‐sufficient in salt production (NI 2024). The Ethiopian government has therefore recommended exploring the potential use of refined salt as a vehicle for folic acid fortification (EPHI 2019). Studies indicate that folic acid can be added to the potassium iodate solution currently used for the Ethiopian national salt iodization program (Modupe and Diosady 2021).

To prepare for possible universal fortification of iodized salt with folic acid, with or without vitamin B_12_, in Ethiopia, additional information is needed on current salt preferences, procurement practices, and utilization, as well consumer acceptability of novel salts fortified with iodine and folic acid (double‐fortified salt, DFS) or iodine, folic acid, and vitamin B_12_ (triple‐fortified salt, TFS) compared to iodized salt (IS) (EPHI 2019). Hence, the objectives of the present study were to assess these issues among Ethiopian WRA residing in rural and urban settings.

## 2 Materials and Methods

### 2.1 Study Locations and Participants

The study was conducted in Gimbichu Woreda, a rural setting in the East Shewa zone of the Oromia region, and in urban Addis Ababa, the nation's capital from August 24, 2022 to September 24, 2022. Gimbichu Woreda was selected as this will be the study site for the follow‐up effectiveness trial using folic acid fortified salt to improve women's folate status. Participant inclusion criteria were: (1) women 15–45 years of age who were in good health and provided informed consent. Exclusion criteria were: (1) currently pregnant or lactating; (2) presence of underlying diseases or medically prescribed salt restriction; and (3) unwillingness to participate.

### 2.2 Sampling Methodology

We employed multi‐stage sampling to select representative samples of participants from two study sites. We obtained enumeration area maps from the Ethiopian Statistical Agency to delineate the selected areas and then compiled a list of all households within each area. We then selected individual households using systematic random sampling from the compiled lists. Through this process 849 households were selected, and, after data cleaning, we report on a total sample of 840 women, 423 in Gimbichu Woreda and 417 in Addis Ababa. The sample size was based on the number of women required to assess contraceptive practices with 5% precision, using a 95% confidence level (Type 1 error of 5%) and 80% power (Type 2 error of 20%). The sample size was calculated using the formula, considering a 97% response rate and a design effect of 1.5. With the available sample size of 840 women, each evaluating all six salts, we have 80% power to detect small pairwise differences of 0.10 standard deviations or greater for the sensorial acceptability and willingness‐to‐use outcome variables.

### 2.3 Data Collection and Management

Information on household demographics, salt preferences, salt procurement practices, salt utilization, and exposure to information about food fortification and neural tube defects (NTDs) was collected from WRA through interactive interviews using a pretested questionnaire. Additionally, we collected data on the consumer acceptability of novel doubly‐fortified salt (DFS) and triply‐fortified salt (TFS) compared with iodized salt (IS). We also gathered data on the willingness to use and purchase the novel salts. The questionnaire was initially prepared in English and then translated into local languages, such as Oromifa (for Gimbuchu) and Amharic (for Addis Ababa). Trained and local language proficient data collectors recorded the survey information using a pre‐tested, automated questionnaire coded in Open Data Kit (ODK) software installed in Techno and Samsung data collection tablets. This system allowed for simultaneous data collection and entry (Hartung et al. 2010), as well as range and consistency checks.

Following each interview, the study participants tasted six different salt samples presented in random order: three coarse salts (C) and three fine salts (F). Each salt type was fortified with iodine only (IS‐C or IS‐F), iodine and folic acid (DFS‐C or DFS‐F), or iodine, folic acid, and vitamin B_12_ (TFS‐C or TFS‐F). The concentration of the fortificant was ~50 ppm for folic acid, 40 ppm for iodine, and 0.48 ppm for vitamin B_12_ in the salts. Salt samples were presented on disposable, rectangular white plates in the respondent's home or compound under natural light, with each salt sample evaluated separately. Participants were instructed to rinse their mouths with water after each test and evaluate the sensory attributes of texture, color, aroma, taste, and overall acceptability using a three‐point hedonic scale (3 = “acceptable and desirable,” 2 = “acceptable, but not desirable,” 1 = “not acceptable”). The data collection tool was designed based on the ten guidelines for sensory assessment suggested by Lawless and Heymann (2010). After sensory testing, respondents were asked if they might use the tested salts and if they would be willing to purchase them at a price equivalent to the current market price of salt in their community.

We transferred data securely to the Ethiopian Public Health Institute server regularly for compilation and review by a dedicated data manager. We identified any missing, incomplete, or implausible data and either corrected or deleted that information from the final data set. When missing data appear in different variables they are excluded from the analysis and are reported in the result sections.

### 2.4 Statistical Analysis

The data analysis involved descriptive statistics to characterize demographics, salt preferences, salt procurement and utilization practices, and willingness to use and purchase the tested salts. We compared the average sensory scores for the six salt types by study area, using one‐way ANOVA with post hoc Duncan test of multiple comparisons. We compared the proportions of respondents who were willing to use and purchase the salts, using pairwise comparisons of the six groups and Z‐tests for independent proportions. We used SPSS version 20 for these analyses.

### 2.5 Ethical Clearance

The Ethiopian Public Health Institute (EPHI) Scientific and Ethical Review Office granted ethical approval for the study (EPHI‐IRB‐354‐2020). We provided comprehensive information to potential participants about the study's purpose and the different salt products to be tested, and we then requested their signed consent to participate.

## 3 Results

### 3.1 Characteristics of Study Participants

Table 1 presents information on the demographic characteristics of the study population. Women in the urban and rural areas were of similar age (*p* > 0.05), but the urban women were more educated and less likely to be married than the rural women (*p* < 0.05). Approximately half of the rural women were non‐literate. Women were much more likely to be responsible for purchasing salt than men in both rural and urban settings (*p* < 0.05), although proportionately more men did so in the rural communities (*p* < 0.05). Knowledge concerning salt iodization and neural tube defects was greater in the urban area than the rural area (*p* < 0.05) (Table 2).

**TABLE 1:** Demographic characteristics of the study population.

Demographic characteristics		Area of residence		
		Rural (*N* = 423)	Urban (*N* = 418)	Total (*N* = 840)
Age of respondents, years (mean ± SD)		31.0 ± 9.4^a^	31.0 ± 8.5^a^	31.0 ± 8.9^a^
Number of people living in the household (mean ± SD)		4.1 ± 1.7^a^	4.0 ± 1.8^a^	4.0 ± 1.8^a^
Marital Status	Married	*N* = 371 (87.7%)^c^	*N* = 291 (69.8%)^a^	*N* = 662 (78.7%)^b^
	Unmarried	*N* = 34 (8.0%)^a^	*N* = 101 (24.3%)^c^	*N* = 135 (16.2%)^b^
	Separated	N = 8 (1.9%)^a^	*N* = 19 (4.5%)^b^	*N* = 27 (3.2%)^a,b^
	Widowed	N = 10 (2.4%)^a^	N = 6 (1.4%)^a^	*N* = 16 (1.9%)^a^
Educational Status	Non‐literate	*N* = 215 (50.8%)^c^	*N* = 39 (9.3%)^a^	*N* = 254 (29.9%)^b^
	No schooling but Can read and write	*N* = 30 (7.2%)^c^	N = 3 (0.7%)^a^	*N* = 33 (4.0%)^b^
	Primary school, Cycle 1 (G: 1–4)	*N* = 55 (13%)^b^	*N* = 35 (8.3%)^a^	*N* = 90 (10.7%)^a,b^
	Primary school, Cycle 2 (G: 5–8)	*N* = 89 (21%)^a^	*N* = 96 (23.1%)^a^	*N* = 185 (22.0%)^a^
	High school	N = 27 (6.3%)^a^	*N* = 175 (41.9%)^c^	*N* = 202 (24.2%)^b^
	Higher education (BSc, MSc, Ph.D.)	*N* = 7 (1.7%)^a^	*N* = 70 (16.7%)^c^	*N* = 77 (9.2%)^b^

*Note:* ^a–c^Any two values in the same row not followed by the same superscript letter are significantly different (*p* < 0.05).

**TABLE 2:** Salt purchasing practices, preferences and information on iodization and NTDs.

Practices related to edible		Area of residence	
		Rural (*N* = 423)	Urban (*N* = 417)
Person purchasing salt for the household (%)	Women	72.8^c,1^	97.4^c,2^
	Men	27.2^b,2^	2.6^b,1^
	Children	0.0^a,1^	0.5^a,1^
Preferred form of salt (%)	Coarse salt	76.1^b,2^	26.8^a,1^
	Fine salt	13.5^a,1^	42.4^b,2^
	Both	10.4^a,1^	30.8^a,2^
Heard about salt iodization (%)		33.7^1^	67.0^2^
Heard about neural tube defects (%)		32.5^1^	46.2^2^

*Note:* ^a–c^Any two values in the same column not followed by the same superscript letter are significantly different (*p* < 0.05). ^1–2^Any two values in the same row not followed by the same superscript number are significantly different (*p* < 0.05).

### 3.2 Salt Preferences and Procurement Practices

**FIGURE 1:**
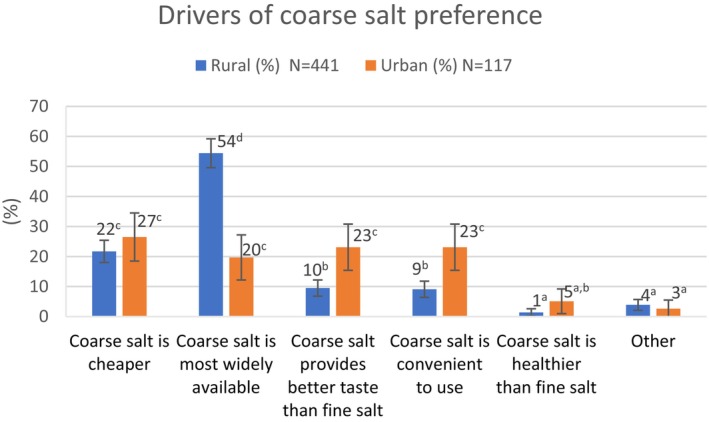
Stated reasons for coarse salt preference in rural and urban settings. ^a–d^Bars not followed by the same superscript letter are significantly different (*p* < 0.05).

**FIGURE 2:**
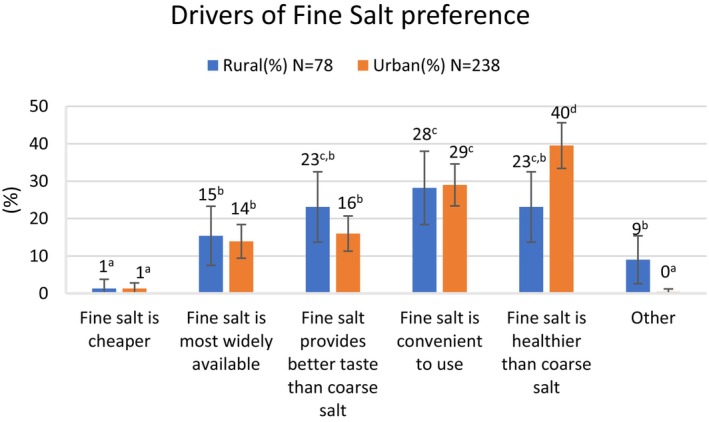
Stated reasons for fine salt preference in rural and urban settings. ^a–d^Bars not followed by the same superscript letter are significantly different (*p* < 0.05).

Rural residents generally preferred coarse salt to fine salt for household consumption, whereas the opposite was true in the city (*p* < 0.05) (Table 2). Thirty percent of urban respondents reported using both types of salt, compared with just 10% of those in the rural communities (*p* < 0.05). Among rural residents who preferred coarse salt, that preference is driven mostly by its greater availability and the fact that it is generally less expensive (Figure 1). Among urban dwellers who preferred coarse salt, their preference is also explained by its lower cost and greater availability, as well as the perception that it is more convenient to use. Rural residents who used fine salt cited its convenience, taste, and a belief that it is healthier, whereas those in the city who used fine salt mainly indicated that they believe fine salt is healthier or more convenient. Salt iodization, suitability of the salt for animal feed, and longer shelf life were rarely reported as reasons for salt preference, so these responses are combined under the category “other” in Figures 1 and 2.

### 3.3 Salt Use Practices

**FIGURE 3:**
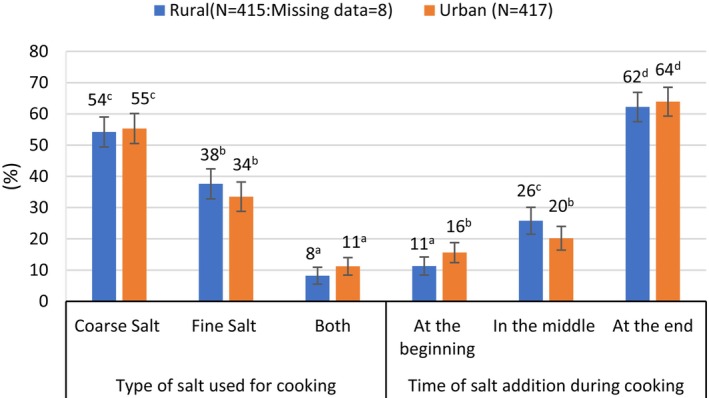
Reported type of salt used for cooking, and timing of salt addition, by area of residence. ^a–d^Bars not followed by the same superscript letter within the same cell are significantly different (*p* < 0.05).

**FIGURE 4:**
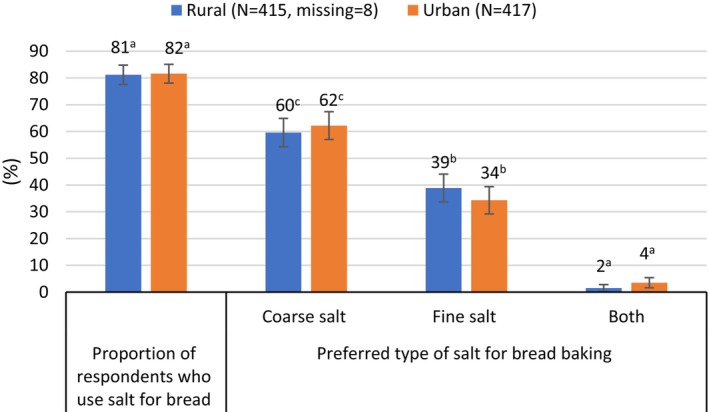
Reported type of salt used for bread making by area of residence. ^a–c^Bars not followed by the same superscript letter within the same cell are significantly different (p < 0.05).

**FIGURE 5:**
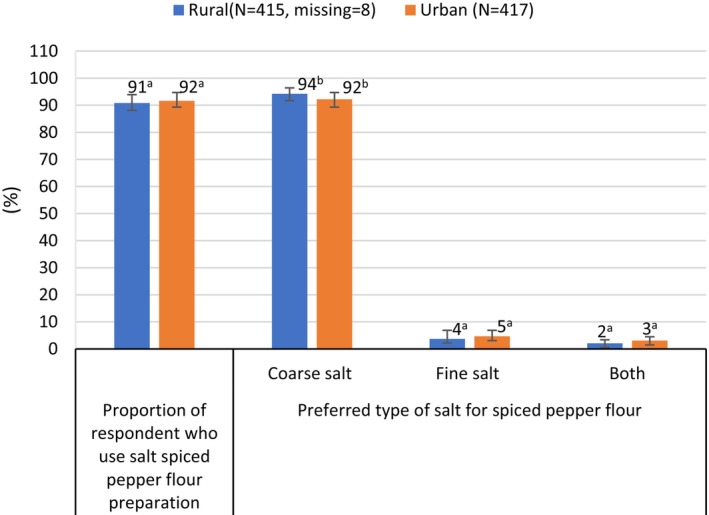
Reported type of salt used for preparing spiced pepper by area of residence. ^a–b^Bars not followed by the same superscript letter within the same cell are significantly different (p < 0.05).

Despite the different reported preferences for coarse or fine salt stated by women in rural versus urban communities, the reported salt utilization practices for cooking, bread making, and preparation of spice mixtures were similar in both sites (Figures 3, 4, 5). All women indicated that they use salt when cooking, 81% stated that they use salt for bread making, 90% reported that they use salt when preparing spiced pepper flour, and 30% of women added salt to mixtures of spiced pea flour. Whereas just over half the women reported using coarse salt for cooking, almost all women reported using coarse salt for bread making and spice mixtures (Figures 3, 4, 5). Salt used for meal preparation was generally added to the cooking pot at the end of cooking (*p* < 0.05) (Figure 3). Although salt is added to coffee in some parts of Ethiopia (Gupta 2023), fewer than 10% of respondents in our study sites stated that they added salt to coffee.

### 3.4 Sensory Assessments

The addition of folic acid to the iodine solution to prepare the DFS changes the color of the salt from white to faint yellow, and the addition of vitamin B_12_ in the folic acid and iodine solution changes the color of the salt to light brown. In our sensory assessments, we compared results separately for coarse and fine salts and for rural versus urban areas. In most cases, the women rated fine salt slightly higher than coarse salt for each attribute, but in all cases the mean rating for all salt attributes was ≥ 2.1, indicating that the salts were considered acceptable (Table 3). There were no differences in assessments of the individual attributes of texture, color, aroma, or taste by site of residence, so the results for urban and rural areas are combined in the table.

**TABLE 3:** Reported acceptability of specific attributes of double‐ and triple‐fortified coarse and fine salts compared with iodized salts.

Sensory characteristics	Study area	*N*	Salt type					
			DFS_F mean ± SD	DFS_C mean ± SD	TFS_F mean ± SD	TFS_C mean ± SD	IDS_F mean ± SD	IDS_C mean ± SD
Texture/form	Total	840	2.6 ± 0.8^1^	2.5 ± 0.8^1^	2.5 ± 0.8^1^	2.5 ± 0.7^1^	2.8 ± 0.5^2^	2.5 ± 0.8^1^
Color	Total	840	2.5 ± 0.8^3^	2.1 ± 0.9^1^	2.6 ± 0.7^3^	2.3 ± 0.9^2^	2.8 ± 0.4^5^	2.7 ± 0.6^4^
Aroma	Total	840	2.8 ± 0.6^3,4^	2.5 ± 0.8^1^	2.7 ± 0.6^3^	2.6 ± 0.7^2^	2.9 ± 0.4^4^	2.8 ± 0.5^3,4^
Taste	Total	840	2.7 ± 0.6^4^	2.4 ± 0.8^1^	2.6 ± 0.7^,3^	2.5 ± 0.8^2^	2.8 ± 0.5^5^	2.7 ± 0.6^4^
Overall acceptability	Rural	423	2.7 ± 0.7^c,2^	2.3 ± 0.9^b,1^	2.6 ± 0.7^b,2^	2.4 ± 0.8^a,1^	2.8 ± 0.5^a,3^	2.6 ± 0.7^a,2^
	Urban	417	2.4 ± 0.8^a,3^	2.1 ± 0.9^a,1^	2.4 ± 0.8^a,3^	2.3 ± 0.9^a,2^	2.8 ± 0.5^a,5^	2.6 ± 0.7^a,4^
	Total	840	2.6 ± 0.8^b,3^	2.2 ± 0.9^ab,1^	2.5 ± 0.8^ab,3^	2.3 ± 0.8^a,2^	2.8 ± 0.5^a,5^	2.6 ± 0.7^a,5^

*Note:* ^a–c^Any two values in the same column not followed by the same superscript letter are significantly different (*p* < 0.05). ^1–5^Any two values in the same row not followed by the same superscript number are significantly different (*p* < 0.05).

**FIGURE 6:**
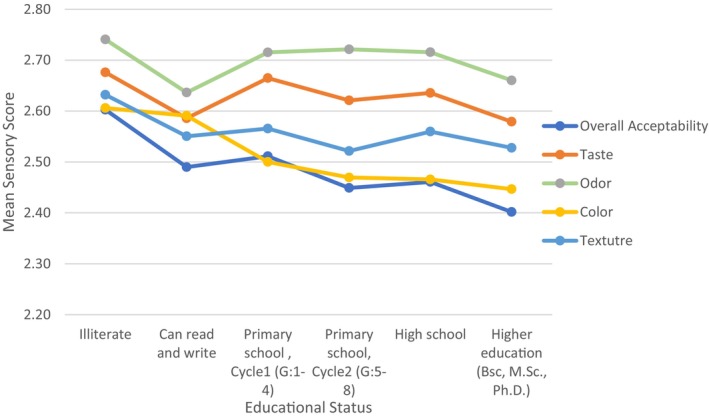
Mean sensory score for fortified salt attributes by educational status.

Figure 6 shows that significant differences in overall acceptability (*p* < 0.05), color (*p* < 0.05), and texture (*p* < 0.05) were observed across educational levels. In contrast, no significant differences were found for taste (*p* > 0.05) and odor (*p* > 0.05).

### 3.5 Willingness to Use and Purchase

The women's reported willingness to use and willingness to purchase the different salt types are presented in Table 4. As with the sensory ratings, there was greater willingness to use and purchase fine salt compared with coarse salt, regardless of the type of fortification or setting. Rural women tended to be more willing than urban women to use and purchase each of the novel salt types. Women in both sites were more willing to use and purchase both types of IS than DFS or TFS, but there was an inconsistent pattern for willingness to use or purchase DFS versus TFS. Both rural and urban women tended to be more willing to use and purchase DFS‐F than TFS‐F, but the reverse was true for DFS‐C versus TFS‐C.

**TABLE 4:** Proportion of respondents reportedly willing to use and purchase the study salts, by site of residence.

Salt type	Willingness to use (%)		Willingness to purchase (%)	
	Rural (*n* = 423)	Urban (*n* = 417)	Rural (*n* = 423)	Urban (*n* = 417)
IS_F	87.7^e,1^	89.7^e,2^	86.8^e,1^	89.7^f,2^
IS_C	80.4^d,2^	76.5^d,1^	79.2^d,2^	76.0^e,1^
DFS_F	80.1^d,2^	65.7^c,1^	79.2^d,2^	65.0^d,1^
DFS_C	59.1^a,2^	50.6^a,1^	57.9^a,2^	49.9^a,1^
TFS_F	74.5^c,2^	65.5^c,1^	73.0^c,2^	63.8^c,1^
TFS_C	61.7^b,2^	58.0^b,1^	61.2^b,2^	57.8^b,1^

*Note:* ^a–e^Any values in the same column not followed by the same superscript letter are significantly different. ^1–2^Any values in the same row for the specified outcome variable not followed by the same superscript number are significantly different.

## 4 Disscusion

In this study we learned that women in rural communities of Ethiopia reportedly prefer coarse salt, whereas those in the urban area prefer fine salt or both coarse and fine salt for household consumption. Despite these differences in stated preferences, women in both settings reported similar patterns of salt types used for cooking, baking, and preparation of spice mixtures. In each of these use cases, coarse salt is reportedly used more commonly than fine salt, mostly because of the greater availability and lower cost of the coarse salt. This observation could have important implications for a future salt fortification program, emphasizing the need to fortify both coarse and fine salt or possibly subsidize the cost of fine salt if only fine salt is fortified.

All types of salt, including novel double‐ and triple‐fortified salts, were generally well accepted during the sensory trials conducted in both rural and urban areas, although currently available iodized salts are generally preferred slightly more than double‐ and triple‐fortified salts, especially with regard to color and texture; and fine salts are preferred to coarse ones. The same preferences are generally reflected in the stated willingness to use and purchase the respective salts, although the reported willingness to use and purchase the multiply‐fortified coarse salt is particularly low. This set of observations implies that introduction of double‐ or triple‐fortified salt is likely to be acceptable, although it will be important to include messaging to explain the added benefits of the DFS and TFS and to overcome possible concerns about color and perceived texture of the novel salts, especially in the urban areas where there seems to be less willingness to purchase the multiply‐fortified salts. These results also suggest that subsidizing the cost of multiply fortified, fine salts could enhance uptake.

The generally positive sensory acceptance of the novel double‐ and triple‐fortified salts observed during sensory trials in both rural and urban areas is promising for public health initiatives primarily aimed at reducing the risk of NTDs. Additionally, these salts hold potential to improve folate status, vitamin B_12_ status and associated morbidities such as macrocytic anemia, extending their public health benefits.

The high consumer acceptability observed in our study aligns with findings from similar studies in diverse settings. For instance, Pattisapu et al. (2024) reported that folic acid–fortified iodized salt was highly acceptable in terms of color and taste among participants in rural India. Similarly, Arynchyna‐Smith et al. (2024) found fortified iodized salt with folic acid to be highly acceptable in terms of taste and color among participants in a metropolitan city in Southern USA, further underscoring the sensory appeal of such products across varying demographic and geographic contexts. Mdoe et al. (2023) demonstrated that quadruple‐fortified salt (QFS: iodine, folate, vitamin B12, and iron) was equally acceptable and had similar sensory scores to standard iodized salt when used to prepare commonly consumed dishes in Tanzania. Additionally, Guetterman et al. (2025) found high acceptability of QFS among women and households in Southern India. Their study noted that rice dishes prepared with QFS and standard iodized salt were indistinguishable based on individual hedonic ratings and household usage patterns.

These findings, coupled with the results of our study, highlight the consistent consumer acceptability of fortified salts with folic acid, and vitamin B_12_ in addition to iodine across diverse cultural, dietary, and geographic settings. This reinforces the feasibility of scaling up double‐ and triple‐fortified salts to improve public health outcomes while maintaining consumer satisfaction.

However, in our study we also found that the slight preference for iodized salts over double‐ and triple‐fortified salts, particularly concerning color, suggests a potential barrier to widespread adoption of fortified salt variants. Incorporating nutrition education alongside the introduction of fortified salts can raise awareness about the crucial roles of folic acid and vitamin B_12_ in preventing neural tube defects, potentially increasing acceptance despite the color differences. In addition, the application of microencapsulation technology for folic acid and vitamin B_12_ fortification of salt in addition to iodine could be explored. This technology offers the possibility of masking color differences and possibly enhancing sensory acceptance, whereas enhancing nutrient stability and enabling controlled release of encapsulated ingredients (Timilsena, Haque, and Adhikari 2020). However, encapsulated fortificants are more costly (Allen et al. 2006) and require different salt processing (mixing) methods, so may not be feasible in Ethiopia without subsidization. Our study also highlights an additional consideration for future fortification programs: since women are the primary salt purchasers for their houshold consumption, nutrition education and marketing interventions should be targeted primarily at them.

On the other hand we found that sensory perception of fortified salt varies across educational levels, with significant declines in overall acceptability, color, and texture ratings among participants with higher education, indicating more critical evaluations. In contrast, taste and odor ratings remained consistent across all education levels, suggesting universal acceptability for these attributes. These findings highlight the potential for fortified salts to be broadly adopted in a universal fortification strategy while emphasizing the need to address specific concerns of highly educated consumers. Tailored messaging focusing on the health benefits and quality of the salts, combined with refinements in sensory attributes like color may be using encapsulation technology, could enhance acceptability across all demographic groups, supporting the successful implementation of fortification programs.

Strengths of the current study include recruitment of both rural and urban populations and a sufficient sample size to detect small differences by salt type and site of residence. Moreover, the use of sensory testing provided empirical data on the acceptibility of the different salts that were being assessed. Limitations of the study are our inability to recruit a nationally representative sample because of funding and logistical limitations. Also, we were not able to include all population sub‐groups, such as children and adult males. We focused on WRA because they are the major target group for folic acid and vitamin B_12_ fortification. It would be helpful to collect information from additional regions of the country before launching a national salt fortification program that includes folic acid and/or vitamin B_12_. In addition, the sensory acceptability of the salts when added to foods was not evaluated as part of the current study. Future research should assess the acceptability of these salts when added to foods.

In conclusion, this study offers valuable insights into the complex factors influencing salt preferences, procurement practices, and sensory evaluations among Ethiopian women. These findings have important implications for the design and implementation of future fortification programs aimed at improving maternal and child health outcomes in Ethiopia. In particular, the double‐ and triple‐fortified salts were generally acceptable. Although coarse salt is currently used more commonly because of its lower cost and greater availability, fine salt was preferred during the sensory testing. Ideally, mandatory fortification of both coarse and fine salt should be considered to reach the largest numbers of consumers. If only fine salt is fortified, subsidization may be critical to ensure widespread uptake. In either case, salt fortification programs designed to provide additional nutrients beyond just iodine should be accompanied by social marketing to create awareness of the nutritional and health benefits of the multiply fortified salt.

## Author Contributions

**Biniyam Tesfaye:** conceptualization (equal), data curation (equal), formal analysis (equal), methodology (equal), validation (equal), visualization (equal), writing original draft (equal), writing – review and editing (equal), Investigation (equal), supervision (equal), funding acquisition (equal), . **Masresha Tessema:** conceptualization (equal), data curation (equal), funding acquisition (equal), investigation (equal), project administration (equal), writing – review and editing (equal). **Charles D. Arnold:** formal analysis (equal), methodology (equal), validation (equal), visualization (equal), writing – review and editing (equal). **Tadesse Kebebe:** data curation (equal), supervision (equal), writing – review and editing (equal). **Tesfaye Zeru:** data curation (equal), supervision (equal), writing – review and editing (equal). **Teshome Assefa:** data curation (equal), supervision (equal), writing – review and editing (equal). **Meseret Woldeyohannes:** data curation (equal), supervision (equal), writing – review and editing (equal). **Getachew Tollera:** conceptualization (equal), funding acquisition (equal), project administration (equal). **Marinus Koning:** conceptualization (equal), funding acquisition (equal), project administration (equal), writing – review and editing (equal). **Homero Martinez:** conceptualization (equal), data curation (equal), funding acquisition (equal), project administration (equal), writing – review and editing (equal). **Christine M. McDonald:** conceptualization (equal), data curation (equal), writing – review and editing (equal). **Kenneth H. Brown:** conceptualization (equal), data curation (equal), writing – original draft (equal), writing – review and editing (equal).

## Data Availability

Data described in the manuscript will be made available upon request through formal application and approval from the Ethiopian Public Health Institute.
